# Piperazine-1,4-diium pyridine-2,3-dicarboxyl­ate methanol monosolvate^1^

**DOI:** 10.1107/S1600536811012384

**Published:** 2011-04-13

**Authors:** Faranak Manteghi, Mohammad Ghadermazi, Nasrin Kakaei

**Affiliations:** a Department of Chemistry, Iran University of Science and Technology, Tehran, Iran; b Department of Chemistry, Faculty of Science, University of Kurdistan, Sanandaj, Iran

## Abstract

The title solvated molecular salt, C_4_H_12_N_2_^2+^·C_7_H_3_NO_4_^2−^·CH_3_OH or (pipzH_2_)(py-2,3-dc)·MeOH, was prepared by the reaction of pyridine-2,3-dicarb­oxy­lic acid (py-2,3-dcH_2_) and piperazine (pipz) in methanol (MeOH) as solvent. One of the two carboxylate groups of the acid fragment is nearly perpendicular to the pyridine ring and the other is almost in its plane [C—C—C—O torsion angles = −85.50 (11) and 88.07 (11)° and N—C—C—O torsion angles = −176.31 (8) and 5.41 (13)°]. In the crystal, the components are linked by O—H⋯O, N—H⋯O and C—H⋯O hydrogen bonds, generating a three-dimensional network.

## Related literature

For similar ion pairs, see: Aghabozorg, Manteghi & Ghadermazi (2008 ▶); Aghabozorg, Manteghi & Sheshmani (2008 ▶). For related metal complexes, see: Barszcz *et al.* (2010 ▶); Li & Li (2004 ▶). 
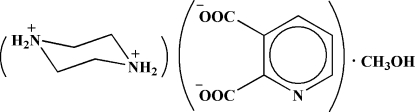


## Experimental

### 

#### Crystal data

C_4_H_12_N_2_^2+^·C_7_H_3_NO_4_^2−^·CH_4_O
*M* *_r_* = 285.30
Monoclinic, 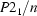

*a* = 8.2541 (6) Å
*b* = 11.8988 (8) Å
*c* = 13.8197 (9) Å
β = 90.288 (2)°
*V* = 1357.27 (16) Å^3^
*Z* = 4
Mo *K*α radiation
μ = 0.11 mm^−1^
*T* = 100 K
0.25 × 0.20 × 0.10 mm

#### Data collection

Bruker SMART APEXII diffractometer
16044 measured reflections
3579 independent reflections
3189 reflections with *I* > 2σ(*I*)
*R*_int_ = 0.023

#### Refinement

*R*[*F* ^2^ > 2σ(*F* ^2^)] = 0.034
*wR*(*F* ^2^) = 0.085
*S* = 1.03
3579 reflections
182 parameters
H-atom parameters constrained
Δρ_max_ = 0.42 e Å^−3^
Δρ_min_ = −0.21 e Å^−3^

### 

Data collection: *APEX2* (Bruker, 2005 ▶); cell refinement: *SAINT* (Bruker, 2005 ▶); data reduction: *SAINT*; program(s) used to solve structure: *SHELXS97* (Sheldrick, 2008 ▶); program(s) used to refine structure: *SHELXL97* (Sheldrick, 2008 ▶); molecular graphics: *PLATON* (Spek, 2009 ▶) and *Mercury* (Macrae *et al.*, 2008 ▶); software used to prepare material for publication: *SHELXL97*.

## Supplementary Material

Crystal structure: contains datablocks I, global. DOI: 10.1107/S1600536811012384/om2416sup1.cif

Structure factors: contains datablocks I. DOI: 10.1107/S1600536811012384/om2416Isup2.hkl

Additional supplementary materials:  crystallographic information; 3D view; checkCIF report

## Figures and Tables

**Table 1:** Hydrogen-bond geometry (Å, °)

*D*—H⋯*A*	*D*—H	H⋯*A*	*D*⋯*A*	*D*—H⋯*A*
N2—H2*A*⋯O4^i^	0.90	1.74	2.6257 (11)	168
N2—H2*B*⋯O2	0.90	1.89	2.7274 (11)	155
N3—H3*A*⋯O1^ii^	0.90	1.85	2.7379 (11)	169
N3—H3*B*⋯O3^iii^	0.90	1.86	2.7393 (11)	166
O5—H5*A*⋯O1	0.85	1.84	2.6867 (10)	171
C3—H3⋯O5^iv^	0.95	2.41	3.3163 (13)	159

Symmetry codes: (i) 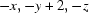; (ii) 
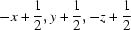
; (iii) 
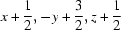
; (iv) 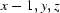.

**Fig. 1.**
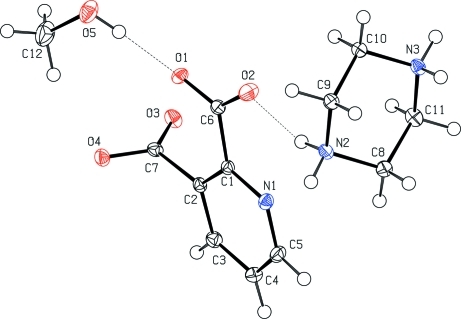
The molecular structure of (pipzH2)(py-2,3-dc).MeOH

**Fig. 2.**
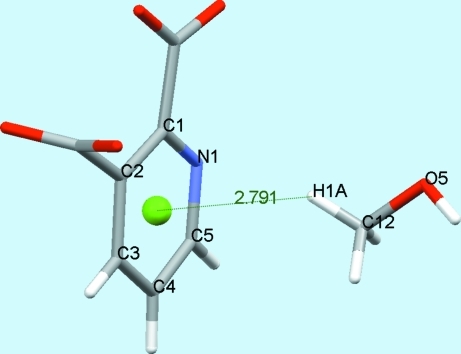
The C–H···π stacking between C12–H1A of methanol and N1/C1-C5 ring of py-2,3-dc.

## supplementary crystallographic information

### Comment

Pyridine-2,3-dicarboxylic acid is remarkably attractive for its flexible and variable coordination modes to construct polymeric architecture. It can act as monodicarboxylate chelating anion, or tridentate coordinating anion with acid hydrogen on nitrogen and doubly deprotonated, tridentate dicarboxylate anion (py-2,3-dc) and even in Mn(II) complexes as tetradenatate and pentadentate ligands (Barszcz *et al.*, 2010; Li & Li, 2004). Preparation of ion pairs with the acid increases the chance of its coordination to metals. Therefore, in our work to obtain proton transfer ion pairs, we have reviewed many ion pairs and their metal complexes (Aghabozorg, Manteghi & Sheshmani, 2008). Also, we have reported a similar ion pair formulated as (pipzH_2_)(pydcH)_2_, (Aghabozorg, Manteghi & Ghadermazi, 2008). The title structure, as shown in Fig. 1, has an asymmetric unit constructed by (pipzH_2_)^2+^ and (py-2,3-dc)^2–^ and a neutral methanol molecule with two hydrogen bonds. There are varieties of other strong and weak hydrogen bonds in the structure, shown in Table 1. Also, a C–O···π stacking, between C6–O2 and N1/C1-C5 ring (-*x*, -*y* + 2, -*z*) with the distance of 3.5240 (9) Å, and a C–H···π stacking, between C12–H1A and N1/C1-C5 ring (*x* +1/2, -*y* +3/2, *z* -1/2), with the distance of 2.791 (1) Å, shown in Fig. 2 are observed.

### Experimental

The title compound was synthesized *via* reaction of 1670 mg (10 mmol) pyridine-2,3-dicarboxylic acid with 860 mg (10 mmol) piperazine in a methanol solution (60 ml). The obtained white precipitate was filtered out and dissolved in water to recrystallize. Colorless crystals of the title compound were obtained after 14 days.

### Refinement

The H(N) and H(O) atoms were found from a difference Fourier map. The H(C) atom positions were calculated. All the hydrogen atoms were refined with isotropic displacement paramaters using a riding model with the *U*_iso_(H) parameters equal to 1.2 times the equivalent isotropic thermal parameter of the bonded C(CH_2_) or N(NH_2_) or 1.5 times that of C(CH_3_) and O(H_2_O). Distances were 0.90 Å for N-H, 0.85 Å for O-H, and 0.95 - 0.99 Å for C-H.

### Crystal data

**Table:**

C_4_H_12_N_2_^2+^·C_7_H_3_NO_4_^2^^−^·CH_4_O	*F*(000) = 608
*M**_r_* = 285.30	*D*_x_ = 1.396 Mg m^−^^3^
Monoclinic, *P*2_1_/*n*	Mo *K*α radiation, λ = 0.71073 Å
Hall symbol: -P 2yn	Cell parameters from 7483 reflections
*a* = 8.2541 (6) Å	θ = 2.3–28.1°
*b* = 11.8988 (8) Å	µ = 0.11 mm^−^^1^
*c* = 13.8197 (9) Å	*T* = 100 K
β = 90.288 (2)°	Prism, colourless
*V* = 1357.27 (16) Å^3^	0.25 × 0.20 × 0.10 mm
*Z* = 4	

### Data collection

**Table:**

Bruker SMART APEXII diffractometer	3189 reflections with *I* > 2σ(*I*)
Radiation source: fine-focus sealed tube	*R*_int_ = 0.023
graphite	θ_max_ = 29.0°, θ_min_ = 2.3°
Detector resolution: 8.3 pixels mm^-1^	*h* = −11→11
φ and ω scans	*k* = −16→16
16044 measured reflections	*l* = −18→18
3579 independent reflections	

### Refinement

**Table:**

Refinement on *F*^2^	Primary atom site location: structure-invariant direct methods
Least-squares matrix: full	Secondary atom site location: difference Fourier map
*R*[*F*^2^ > 2σ(*F*^2^)] = 0.034	Hydrogen site location: difference Fourier map
*wR*(*F*^2^) = 0.085	H-atom parameters constrained
*S* = 1.03	*w* = 1/[σ^2^(*F*_o_^2^) + (0.035*P*)^2^ + 0.650*P*] where *P* = (*F*_o_^2^ + 2*F*_c_^2^)/3
3579 reflections	(Δ/σ)_max_ = 0.001
182 parameters	Δρ_max_ = 0.42 e Å^−^^3^
0 restraints	Δρ_min_ = −0.21 e Å^−^^3^

### Special details

**Table:**

Geometry. All e.s.d.'s (except the e.s.d. in the dihedral angle between two l.s. planes) are estimated using the full covariance matrix. The cell e.s.d.'s are taken into account individually in the estimation of e.s.d.'s in distances, angles and torsion angles; correlations between e.s.d.'s in cell parameters are only used when they are defined by crystal symmetry. An approximate (isotropic) treatment of cell e.s.d.'s is used for estimating e.s.d.'s involving l.s. planes.
Refinement. Refinement of *F*^2^ against ALL reflections. The weighted *R*-factor *wR* and goodness of fit *S* are based on *F*^2^, conventional *R*-factors *R* are based on *F*, with *F* set to zero for negative *F*^2^. The threshold expression of *F*^2^ > σ(*F*^2^) is used only for calculating *R*-factors(gt) *etc*. and is not relevant to the choice of reflections for refinement. *R*-factors based on *F*^2^ are statistically about twice as large as those based on *F*, and *R*- factors based on ALL data will be even larger.

### Fractional atomic coordinates and isotropic or equivalent isotropic displacement parameters (Å^2^)

**Table:**

	*x*	*y*	*z*	*U*_iso_*/*U*_eq_	
O1	0.16285 (8)	0.76486 (6)	0.03344 (5)	0.01401 (15)	
O2	0.21626 (9)	0.91599 (6)	0.12414 (6)	0.01918 (16)	
O3	−0.13943 (9)	0.61040 (6)	0.03291 (5)	0.01666 (16)	
O4	−0.11791 (9)	0.70279 (6)	−0.10648 (5)	0.01627 (15)	
N1	−0.09833 (10)	0.97903 (7)	0.11968 (6)	0.01339 (17)	
N2	0.17152 (10)	1.11819 (7)	0.21302 (6)	0.01264 (16)	
H2A	0.1410	1.1746	0.1735	0.015*	
H2B	0.1538	1.0523	0.1828	0.015*	
N3	0.31476 (10)	1.05117 (7)	0.39424 (6)	0.01304 (16)	
H3A	0.3349	1.1189	0.4206	0.016*	
H3B	0.3473	0.9987	0.4371	0.016*	
C1	−0.05754 (11)	0.88142 (8)	0.07675 (6)	0.01078 (17)	
C2	−0.17210 (11)	0.80866 (8)	0.03586 (7)	0.01165 (18)	
C3	−0.33567 (12)	0.83780 (9)	0.04316 (7)	0.01506 (19)	
H3	−0.4169	0.7896	0.0175	0.018*	
C4	−0.37860 (12)	0.93756 (9)	0.08809 (7)	0.0166 (2)	
H4	−0.4891	0.9585	0.0943	0.020*	
C5	−0.25545 (12)	1.00615 (9)	0.12379 (7)	0.01563 (19)	
H5	−0.2845	1.0758	0.1525	0.019*	
C6	0.12243 (11)	0.85332 (8)	0.07816 (7)	0.01183 (18)	
C7	−0.13540 (11)	0.69936 (8)	−0.01603 (7)	0.01243 (18)	
C8	0.07828 (12)	1.12692 (9)	0.30451 (7)	0.01538 (19)	
H8A	−0.0383	1.1149	0.2908	0.018*	
H8B	0.0914	1.2032	0.3320	0.018*	
C9	0.34843 (12)	1.13038 (8)	0.23146 (7)	0.01367 (18)	
H9A	0.3709	1.2059	0.2584	0.016*	
H9B	0.4080	1.1231	0.1698	0.016*	
C10	0.40670 (12)	1.04139 (8)	0.30194 (7)	0.01438 (19)	
H10A	0.3901	0.9658	0.2737	0.017*	
H10B	0.5239	1.0513	0.3148	0.017*	
C11	0.13684 (12)	1.04015 (9)	0.37760 (7)	0.0162 (2)	
H11A	0.0790	1.0508	0.4395	0.019*	
H11B	0.1121	0.9637	0.3533	0.019*	
O5	0.39165 (9)	0.71135 (8)	−0.09625 (6)	0.02485 (19)	
H5A	0.3255	0.7344	−0.0537	0.037*	
C12	0.29401 (15)	0.65794 (12)	−0.16692 (9)	0.0302 (3)	
H1A	0.3427	0.6679	−0.2309	0.045*	
H1B	0.1855	0.6913	−0.1667	0.045*	
H1C	0.2861	0.5776	−0.1522	0.045*	

### Atomic displacement parameters (Å^2^)

**Table:**

	*U*^11^	*U*^22^	*U*^33^	*U*^12^	*U*^13^	*U*^23^
O1	0.0156 (3)	0.0103 (3)	0.0162 (3)	0.0017 (2)	0.0019 (3)	−0.0011 (3)
O2	0.0139 (3)	0.0188 (4)	0.0248 (4)	0.0001 (3)	−0.0031 (3)	−0.0083 (3)
O3	0.0236 (4)	0.0110 (3)	0.0153 (3)	−0.0009 (3)	−0.0041 (3)	0.0011 (3)
O4	0.0233 (4)	0.0130 (3)	0.0124 (3)	0.0028 (3)	−0.0019 (3)	−0.0007 (3)
N1	0.0154 (4)	0.0117 (4)	0.0130 (4)	0.0005 (3)	0.0011 (3)	−0.0010 (3)
N2	0.0152 (4)	0.0111 (4)	0.0116 (4)	0.0003 (3)	−0.0022 (3)	0.0000 (3)
N3	0.0168 (4)	0.0105 (4)	0.0118 (4)	−0.0007 (3)	−0.0025 (3)	0.0007 (3)
C1	0.0128 (4)	0.0103 (4)	0.0093 (4)	0.0003 (3)	0.0007 (3)	0.0009 (3)
C2	0.0143 (4)	0.0109 (4)	0.0097 (4)	0.0003 (3)	−0.0006 (3)	0.0008 (3)
C3	0.0135 (4)	0.0173 (5)	0.0144 (4)	−0.0005 (3)	−0.0013 (3)	−0.0005 (4)
C4	0.0134 (4)	0.0201 (5)	0.0163 (4)	0.0037 (4)	0.0002 (3)	0.0002 (4)
C5	0.0181 (5)	0.0136 (4)	0.0152 (4)	0.0032 (4)	0.0023 (3)	−0.0016 (3)
C6	0.0132 (4)	0.0111 (4)	0.0112 (4)	0.0005 (3)	0.0006 (3)	0.0015 (3)
C7	0.0117 (4)	0.0115 (4)	0.0141 (4)	0.0001 (3)	−0.0035 (3)	−0.0015 (3)
C8	0.0142 (4)	0.0172 (5)	0.0148 (4)	0.0009 (4)	0.0003 (3)	0.0002 (4)
C9	0.0142 (4)	0.0137 (4)	0.0132 (4)	−0.0004 (3)	−0.0002 (3)	0.0007 (3)
C10	0.0154 (4)	0.0146 (4)	0.0130 (4)	0.0021 (3)	−0.0011 (3)	−0.0002 (3)
C11	0.0160 (5)	0.0168 (5)	0.0159 (4)	−0.0040 (4)	−0.0006 (3)	0.0028 (4)
O5	0.0159 (4)	0.0345 (5)	0.0242 (4)	−0.0027 (3)	0.0035 (3)	−0.0117 (3)
C12	0.0239 (6)	0.0398 (7)	0.0269 (6)	−0.0016 (5)	0.0002 (5)	−0.0160 (5)

### Geometric parameters (Å, °)

**Table:**

O1—C6	1.2662 (12)	C3—H3	0.9500
O2—C6	1.2469 (12)	C4—C5	1.3920 (14)
O3—C7	1.2566 (12)	C4—H4	0.9500
O4—C7	1.2597 (12)	C5—H5	0.9500
N1—C5	1.3379 (13)	C8—C11	1.5215 (14)
N1—C1	1.3477 (12)	C8—H8A	0.9900
N2—C8	1.4871 (12)	C8—H8B	0.9900
N2—C9	1.4881 (12)	C9—C10	1.5155 (13)
N2—H2A	0.9001	C9—H9A	0.9900
N2—H2B	0.9001	C9—H9B	0.9900
N3—C11	1.4911 (13)	C10—H10A	0.9900
N3—C10	1.4920 (12)	C10—H10B	0.9900
N3—H3A	0.9000	C11—H11A	0.9900
N3—H3B	0.9001	C11—H11B	0.9900
C1—C2	1.3992 (13)	O5—C12	1.4139 (14)
C1—C6	1.5227 (13)	O5—H5A	0.8500
C2—C3	1.3981 (13)	C12—H1A	0.9800
C2—C7	1.5164 (13)	C12—H1B	0.9800
C3—C4	1.3864 (14)	C12—H1C	0.9800
			
C5—N1—C1	118.09 (8)	O4—C7—C2	117.75 (8)
C8—N2—C9	111.05 (7)	N2—C8—C11	110.67 (8)
C8—N2—H2A	108.6	N2—C8—H8A	109.5
C9—N2—H2A	107.6	C11—C8—H8A	109.5
C8—N2—H2B	111.9	N2—C8—H8B	109.5
C9—N2—H2B	108.8	C11—C8—H8B	109.5
H2A—N2—H2B	108.9	H8A—C8—H8B	108.1
C11—N3—C10	111.47 (7)	N2—C9—C10	110.48 (8)
C11—N3—H3A	108.7	N2—C9—H9A	109.6
C10—N3—H3A	108.9	C10—C9—H9A	109.6
C11—N3—H3B	109.3	N2—C9—H9B	109.6
C10—N3—H3B	110.9	C10—C9—H9B	109.6
H3A—N3—H3B	107.5	H9A—C9—H9B	108.1
N1—C1—C2	122.77 (9)	N3—C10—C9	109.50 (8)
N1—C1—C6	115.44 (8)	N3—C10—H10A	109.8
C2—C1—C6	121.77 (8)	C9—C10—H10A	109.8
C3—C2—C1	117.92 (9)	N3—C10—H10B	109.8
C3—C2—C7	116.24 (8)	C9—C10—H10B	109.8
C1—C2—C7	125.84 (8)	H10A—C10—H10B	108.2
C4—C3—C2	119.59 (9)	N3—C11—C8	110.61 (8)
C4—C3—H3	120.2	N3—C11—H11A	109.5
C2—C3—H3	120.2	C8—C11—H11A	109.5
C3—C4—C5	118.22 (9)	N3—C11—H11B	109.5
C3—C4—H4	120.9	C8—C11—H11B	109.5
C5—C4—H4	120.9	H11A—C11—H11B	108.1
N1—C5—C4	123.36 (9)	C12—O5—H5A	104.9
N1—C5—H5	118.3	O5—C12—H1A	109.5
C4—C5—H5	118.3	O5—C12—H1B	109.5
O2—C6—O1	125.55 (9)	H1A—C12—H1B	109.5
O2—C6—C1	118.59 (8)	O5—C12—H1C	109.5
O1—C6—C1	115.84 (8)	H1A—C12—H1C	109.5
O3—C7—O4	124.39 (9)	H1B—C12—H1C	109.5
O3—C7—C2	117.52 (8)		
			
C5—N1—C1—C2	0.66 (14)	N1—C1—C6—O1	−176.31 (8)
C5—N1—C1—C6	−177.60 (8)	C2—C1—C6—O1	5.41 (13)
N1—C1—C2—C3	−2.12 (14)	C3—C2—C7—O3	−85.50 (11)
C6—C1—C2—C3	176.03 (8)	C1—C2—C7—O3	94.23 (12)
N1—C1—C2—C7	178.16 (9)	C3—C2—C7—O4	88.07 (11)
C6—C1—C2—C7	−3.69 (14)	C1—C2—C7—O4	−92.20 (12)
C1—C2—C3—C4	1.40 (14)	C9—N2—C8—C11	−56.46 (10)
C7—C2—C3—C4	−178.84 (9)	C8—N2—C9—C10	58.30 (10)
C2—C3—C4—C5	0.61 (15)	C11—N3—C10—C9	57.68 (10)
C1—N1—C5—C4	1.56 (15)	N2—C9—C10—N3	−58.17 (10)
C3—C4—C5—N1	−2.20 (15)	C10—N3—C11—C8	−56.39 (11)
N1—C1—C6—O2	5.41 (13)	N2—C8—C11—N3	55.14 (11)
C2—C1—C6—O2	−172.86 (9)		

### Hydrogen-bond geometry (Å, °)

**Table:**

*D*—H···*A*	*D*—H	H···*A*	*D*···*A*	*D*—H···*A*
N2—H2A···O4^i^	0.90	1.74	2.6257 (11)	168
N2—H2B···O2	0.90	1.89	2.7274 (11)	155
N3—H3A···O1^ii^	0.90	1.85	2.7379 (11)	169
N3—H3B···O3^iii^	0.90	1.86	2.7393 (11)	166
O5—H5A···O1	0.85	1.84	2.6867 (10)	171
C3—H3···O5^iv^	0.95	2.41	3.3163 (13)	159

Symmetry codes: (i) −*x*, −*y*+2, −*z*;  (ii) −*x*+1/2, *y*+1/2, −*z*+1/2; (iii) *x*+1/2, −*y*+3/2, *z*+1/2; (iv) *x*−1, *y*, *z*.
